# Juvenile idiopathic arthritis: the transition to adulthood

**DOI:** 10.1186/1546-0096-12-S1-P193

**Published:** 2014-09-17

**Authors:** Susana Fernandes, José António Melo Gomes, Sónia Melo Gomes, Fernando Martins

**Affiliations:** 1 Rheumatology, Instituto Portugues De Reumatologia, Lisbon, Portugal; 2 Bioinformatician, Sociedade Portuguesa de Reumatologia, Lisboa, Portugal

## Introduction

Juvenile Idiopathic Arthritis (JIA) is term used to classify a group of heterogeneous pediatric rheumatic diseases. Many of these conditions remain active until adulthood and when patients start to be followed by adult Rheumatologists there may arise some classification problems once AIA (Adult Idiopatic Arthritis) does not exist! Many published papers regarding the transition of JIA into adulthood miss this point.

## Objectives

Our aim is to analyze the characteristics of 206 JIA patients, currently in their adulthood, that have been followed, in most of their disease, by the same Rheumatologist with a follow-up time superior to 30 years in some cases.

## Methods

This study includes 206 patients currently in adult age from a sample of 369 JIA patients, continuously followed by the first author in the Children, Adolescent and Young Adult Rheumatology Outpatients Clinic at IPR and Private Practice. All these patients are registered in REUMA.PT, the National Registry for rheumatic diseases of the Sociedade Portuguesa de Reumatologia. The 2010 EULAR/ACR Criteria1 for the classification of RA and the ASAS Criteria for Classification of Axial2 and Peripheral3 Spondyloarthritis were used.

## Results

The group included 126 female and 80 male patients, with a mean age of 30.0 +/-11.0 years, having mean disease duration of 21.5 +/-11.3 years. The presentation forms and definitive diagnosis are listed below. Sixty three of these patients are in complete and prolonged off therapy remission. 112 patients were treated with methotrexate, 42 are or were previously treated with biological agents, and 33 had been subjected to intra-articular injections (triamcinolone hexacetonide). Other aspects concerning therapy, morbidity and mortality were also analyzed. All of these patients are registered in SPR database (REUMA.PT).

## Conclusion

It’s clear that JIA is a group of several joint diseases that start in children and may continue to affect these patients throughout their adult life. A significative group of this patients can be classified as **juvenile spondyloarthritis** (75/206 = 36%) This analysis shows that JIAs are not a benign and self-limiting disease group, being essential to ensure the proper continuity of rheumatologic care for these patients in adulthood, preferably using a common language and approach to classify and treat these patients.

## Disclosure of interest

None declared.

**Table 1:** 

Initial presentation	Number of patients	Current diagnosis	Number of patients
Oligo persistent	58	Still's Disease	34
Oligo extended	31	Rheumatoid Arthritis	24
Poli FRIgM +	17	Axial Spondylarthritis	14
Poli FRIgM -	23	Perypheral Spondylarthritis	39
Systemic	34	Reactive arthritis	2
Arthritis/Entesitis	30	Psoriatic Arthritis	9
Psoriatic arthritis	7	Inflammatory Bowel Disease Arthropathy	11
Inflammatory bowel disease	6	Oligo/ANA+ with Chronic Uveitis	18
		
**Ocular manifestations**		Other	16
		
***Uveitis***	**25** [22 Chronic + 3 Acute]	Without definitive diagnosis	39

